# Transcriptome Analysis of *Beta macrocarpa* and Identification of Differentially Expressed Transcripts in Response to *Beet Necrotic Yellow Vein Virus* Infection

**DOI:** 10.1371/journal.pone.0132277

**Published:** 2015-07-21

**Authors:** Huiyan Fan, Yongliang Zhang, Haiwen Sun, Junying Liu, Ying Wang, Xianbing Wang, Dawei Li, Jialin Yu, Chenggui Han

**Affiliations:** 1 State Key Laboratory for Agrobiotechnology and Department of Plant Pathology, China Agricultural University, Beijing, 100193, China; 2 College of Pharmacy, Zhejiang Chinese Medicine University, Hangzhou, 310053, Zhejiang, China; Uppsala University, SWEDEN

## Abstract

**Background:**

Rhizomania is one of the most devastating diseases of sugar beet. It is caused by *Beet necrotic yellow vein virus* (BNYVV) transmitted by the obligate root-infecting parasite *Polymyxa betae*. *Beta macrocarpa*, a wild beet species widely used as a systemic host in the laboratory, can be rub-inoculated with BNYVV to avoid variation associated with the presence of the vector *P*. *betae*. To better understand disease and resistance between beets and BNYVV, we characterized the transcriptome of *B*. *macrocarpa* and analyzed global gene expression of *B*. *macrocarpa* in response to BNYVV infection using the Illumina sequencing platform.

**Results:**

The overall *de novo* assembly of cDNA sequence data generated 75,917 unigenes, with an average length of 1054 bp. Based on a BLASTX search (E-value ≤ 10^−5^) against the non-redundant (NR, NCBI) protein, Swiss-Prot, the Gene Ontology (GO), Clusters of Orthologous Groups of proteins (COG) and Kyoto Encyclopedia of Genes and Genomes (KEGG) databases, there were 39,372 unigenes annotated. In addition, 4,834 simple sequence repeats (SSRs) were also predicted, which could serve as a foundation for various applications in beet breeding. Furthermore, comparative analysis of the two transcriptomes revealed that 261 genes were differentially expressed in infected compared to control plants, including 128 up- and 133 down-regulated genes. GO analysis showed that the changes in the differently expressed genes were mainly enrichment in response to biotic stimulus and primary metabolic process.

**Conclusion:**

Our results not only provide a rich genomic resource for beets, but also benefit research into the molecular mechanisms of beet- BNYV Vinteraction.

## Introduction

Rhizomania is a major threat to sugar beet (*Beta vulgaris*) production, through reductions in both crop yield and beet sugar content, and is distributed worldwide across regions depending on cultivation conditions [1,2]. It is caused by *Beet necrotic yellow vein virus* (BNYVV), which is transmitted by the obligate root-infecting parasite *Polymyxa betae* in a persistent manner because of resting spores retaining the virus [3]. BNYVV has a multipartite RNA genome, the larger RNA1 and RNA2 contain the housekeeping genes of the virus and are required for infection, whereas the smaller RNAs are involved in pathogenicity and vector transmission [4–6]. Up to now, the major focus in study of the host–pathogen interaction with BNYVV has been on the virus itself, and there have been very few studies of the host genomic response. Recently, gene expression profiling using Illumina RNA-Seq revealed 3,016 differentially expressed genes during BNYVV infection in the experimental host *Nicotiana benthamiana*, which provided a list of candidate genes involved in resistance to BNYVV infection [7]. However, RNA-Seq technology has not been used to analyze the natural hosts’ genomic response to BNYVV infection.

Because of the lack of effective and acceptable chemical control methods for the vector, genetic resistance is the most promising approach for control of this disease [1]. A number of different sources of partial resistance to BNYVV have been developed and these have performed better than susceptible cultivars in the field [8–10]. However, this breeding success may be a short lived as the presence of virulent forms of BNYVV, such as the P-type, and resistance-breaking isolates suggest that further research is required to identity new sources of resistance for breeders to work with [11,12]. The recent genome sequencing of sugar beet provided a powerful tool to characterize the genes responsible for agronomic traits [13]. However, the transcriptome of only one species may not provide an understanding of gene/genome evolution, genome organization and genetic variations. Thus, sequencing the transcriptome of wild species should lead toward better understanding of evolutionary processes and knowledge about the impact of gene flow to domesticated plants [14]. In addition, wild species could be new sources of resistance genes. The Holly resistance source has been widely exploited in most current varieties. It is well known that BNYVV resistance genes are derived from wild beet *B*. *vulgaris* subsp. *maritime* accessions [8,15].

Very few genomic resources are available for wild beet relatives; and this includes *B*. *macrocarpa*, a widely used laboratory systemic host, which can be rub-inoculated with BNYVV, thereby avoiding variation associated with presence of the vector *P*. *betae*. Only nine related nucleotide sequences were available for *B*. *macrocarpa* in GenBank as of June 2014. Next generation sequencing technologies have enabled very rapid and cost-effective sequencing of transcriptomes. In this study, we took advantage of RNA-Seq to survey the foliar transcriptome of *B*. *macrocarpa* and identified a total 75,917 unigenes. Comparative analysis of the expression profiles of BNYVV-infected and non-infected *B*. *macrocarpa* indicated that BNYVV infection could potentially perturb primary metabolism of plants and activate immune regulatory systems. To our knowledge, this is the first report to define the *B*. *macrocarpa* transcriptome and could enrich the genetic resources of beets and aid in discovery of genes related to the molecular mechanism of beet–BNYVV interactions.

## Results and Discussion

### Illumina paired-end sequencing and *de novo* assembly

**Fig 1:**
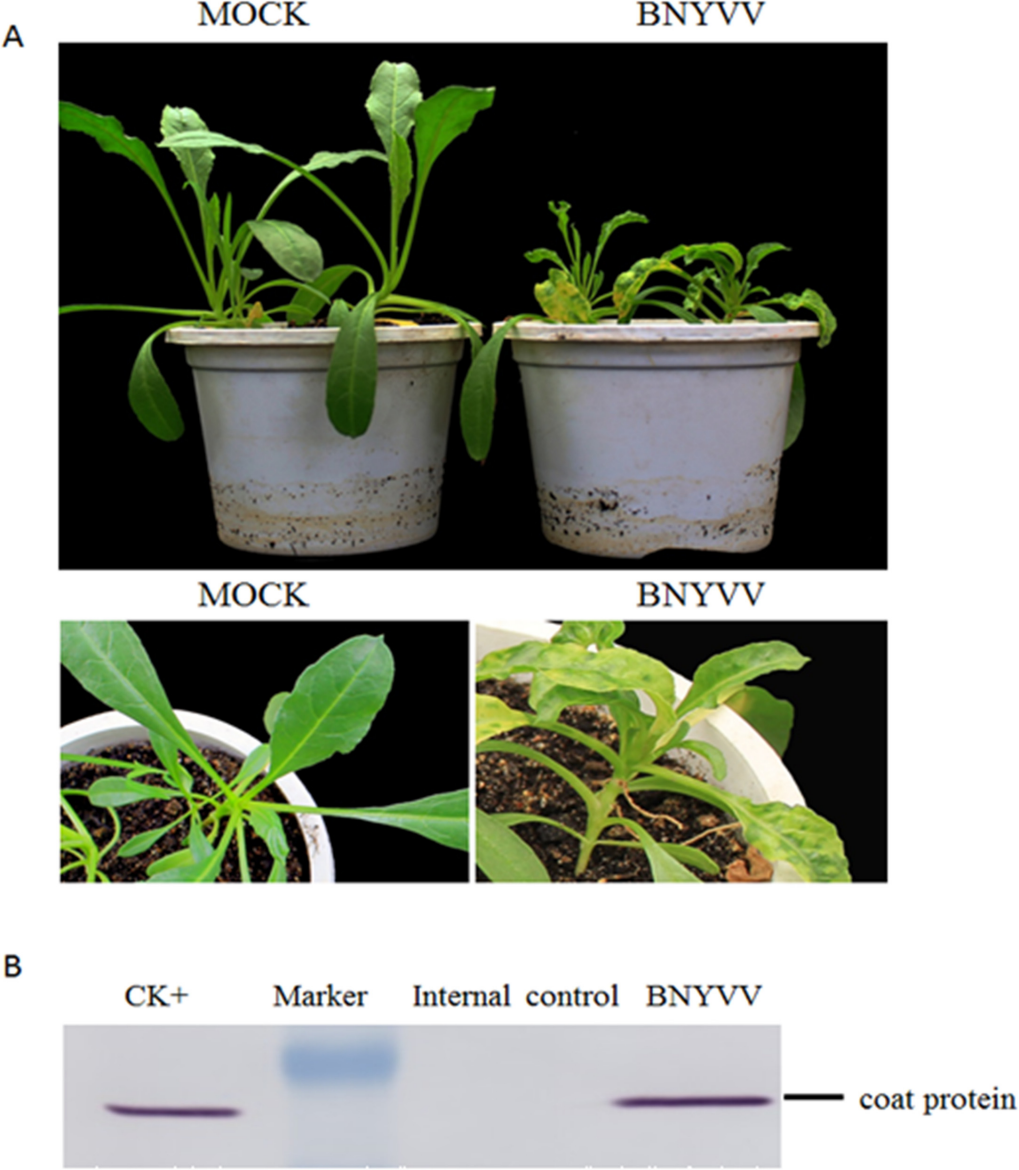
BNYVV phenotypes and detection of virus in systemically infected *B*. *macrocarpa* leaves. (A) Systemic symptoms induced in *B*. *macrocarpa* plants by BNYVV. Mock-infection was plants rubbed with buffer. Mock and BNYVV (B) Western blotting for analysis of BNYVV CP expression. BNYVV-infected leaves of *Tetragonia expansa* were the positive control (CK^+^ lane) and healthy *B*. *macrocarpa* leaves (mock) were the negative control.

Under greenhouse conditions, the leaves of *B*. *macrocarpa* inoculated with BNYVV showed chlorotic spots at 5–7 d, followed by systemic yellow mosaic with severe stunting after two weeks (Fig 1). To obtain an overview of *B*. *macrocarpa* gene expression profile and genes responding to virus in leaves, we prepared two respective RNA samples from the leaf pools of BNYVV-infected and non-infected plants for transcriptome analysis using the Illumina sequencing platform Hiseq2000.

Paired-end sequencing resulted in a total of 121,269,730 reads comprising 12,126,973,000 nucleotide bases. To improve quality of the dataset, high stringency filtering was performed, which included removal of reads containing adaptor and vector sequence as well as reads with 30% having base quality ≤ 20. There were 48,279,346 and 59,903,306 clean reads from BNYVV-infected and non-infected libraries (S1 Table). The raw reads are available in the Sequence Read Archive at the National Center for Biotechnology Information (NCBI) under accession number SRP033294. Using the SOAPdenovo assembly program, all high-quality reads were assembled into 44,626 and 44,903 contigs, with N50s (N50 represents median length of all contigs) of 545 and 520 bp, respectively. CAP3 software[16] allowed us to map the reads back to the contigs and connect the contigs into unigenes with the fewest Ns (N represents unknown residues between two contigs) and could not be extended on either end. Finally, we obtained a total of 75,917 unigenes, with a N50 length of 1867 bp and total length of 80.0 Mb (Table 1). The length distribution of non-redundant unigenes is shown in Table 2, implying that the Illumina sequencing solution was reliable.

**Table 1:** Overview of the sequencing and assembly.

Statistics of data production	Mock	BNYVV infected
Clean reads	48,279,346	59,903,306
Clean bases (bp)	4,693,645,595	5,830,613,062
Contigs		
No. of contigs	44,626	44,903
Total nucleotides(nt) in contigs	21,231,334	20,853,654
Length of N50 (bp)	545	520
Average length of contigs (bp)	476	464
GC percentage (%)	41.03	41.00
All-Unigenes		
No. of All-unigenes	75,917	
Total nucleotides(nt) in All-unigenes	79,982,383	
Length of N50 (bp)	1,867	
Average length of unigenes (bp)	1,054	
GC percentage (%)	39.82	

**Table 2:** Length distribution of unigenes in the assembled transcriptomes.

Unigene length (bp)	Total number	Percentage (%)
0–500	34368	45.27
500–1000	13751	18.11
1000–1500	8613	11.35
1500–2000	7124	9.38
2000–3000	7457	9.82
3000–4000	2966	3.91
4000–5000	954	1.26
5000–10000	660	0.87
≥10000	24	0.03
Total	75917	100.00

### Similarity analysis and functional annotation

**Fig 2:**
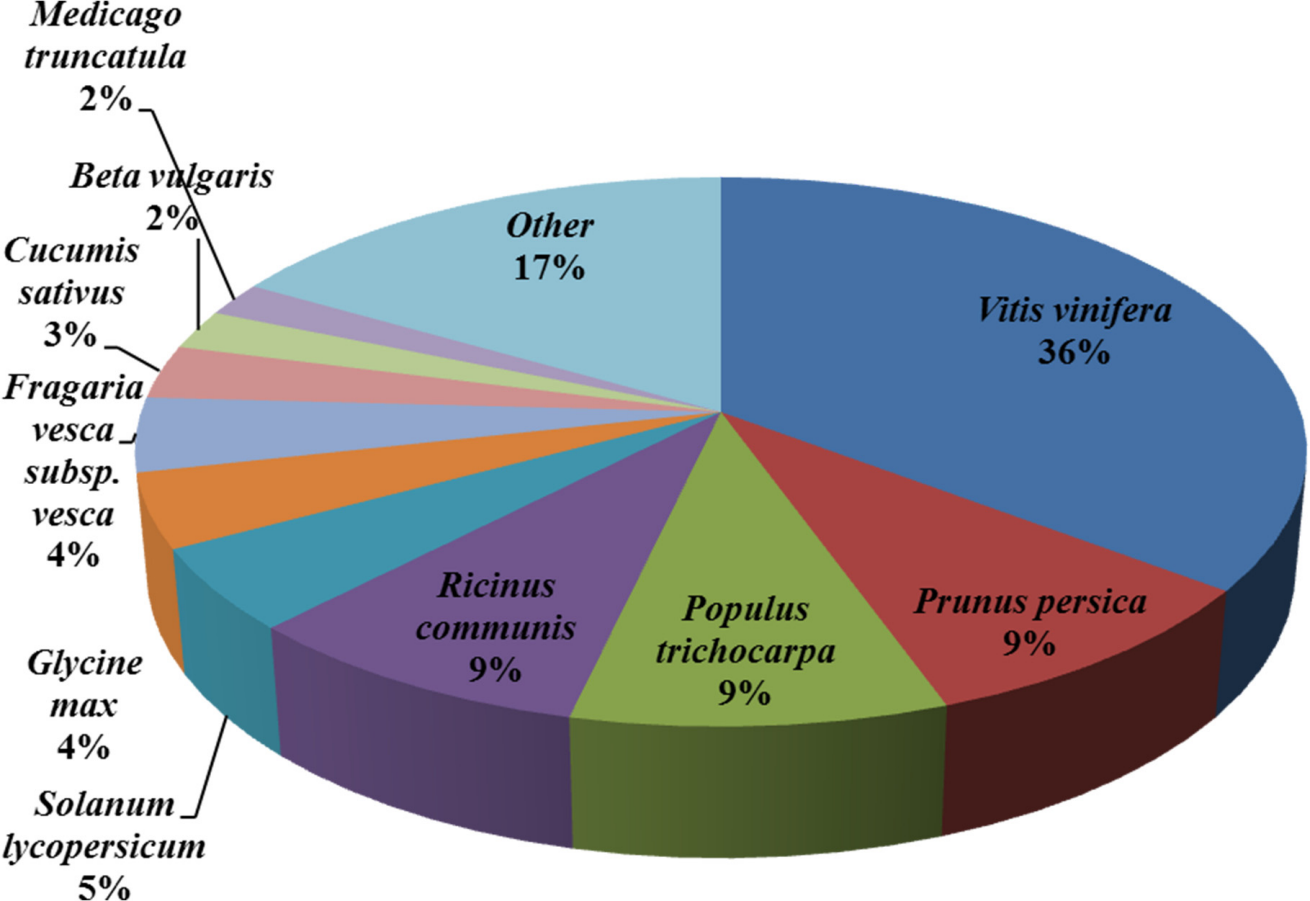
Species distribution of unigene BLASTX results against the NCBI NR protein database, with a cut-off E-value ≤ 10^−5^ and proportions of each unigene species. Different colors represent different species.

All unigenes were compared with the NCBI non-redundant (NR) protein and Swiss-Prot databases for functional annotation using BLASTX with an E-value threshold of 10^−5^ (E-value ≤ 10^−5^). Among the 75,917 unigenes from both the BNYVV-infected and non-infected libraries, 39,176 (51.60%) showed significant matches in the NR database. An additional 26,326 (34.68%) unigenes showed significant matches in the Swiss-Prot database (Table 3, S1 File). The species distribution of the best match result for each sequence is shown in Fig 2. The sequences of *B*. *macrocarpa* had a 36% match with grape (*Vitis vinifera*), followed by flowering peach (*Prunus persica*), white poplar (*Populus trichocarpa*) and castor (*Ricinus communis*) with 9%. In addition, there were 784 distinct sequences with the highest homology to genes from *B*. *vulgaris*, supporting the robustness and validity of our RNA-Seq based approach. The sequencing in this study provides more detail and general genetic data that will facilitate large-scale discovery and utilization of genetic resources for *B*. *macrocarpa*.

**Table 3:** Summary of functional annotation of all B. macrocarpa unigenes.

Database	75,917 all unigenes	
	Number of annotated sequences	Percentage of annotated sequences (%)
NR	39176	51.60
SwissProt	26326	34.68
KEGG	7209	9.50
COG	14116	18.59
GO	16332	21.51

**Fig 3:**
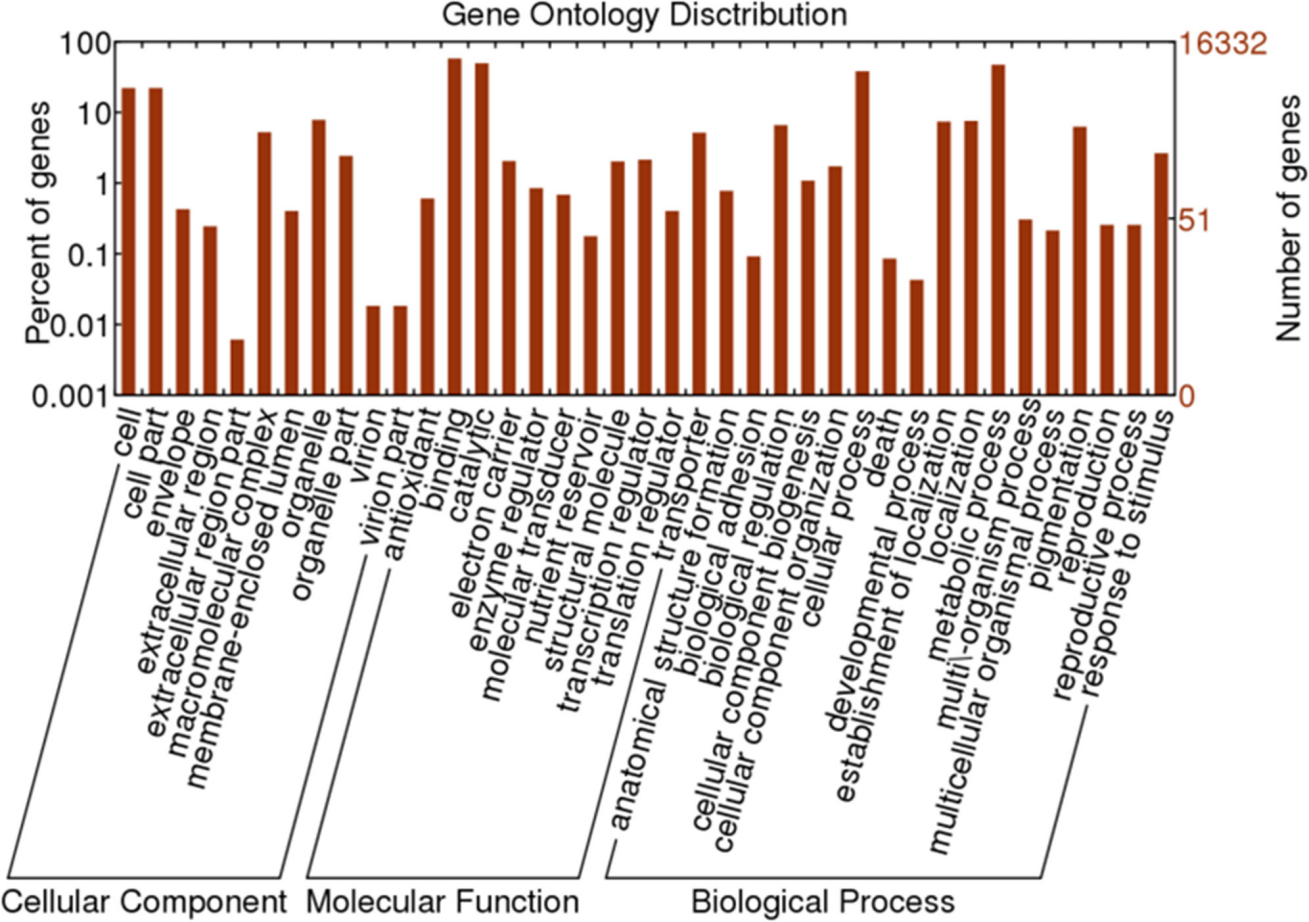
Gene ontology classification of *B*. *macrocarpa* leaves transcriptome. The functions of unigenes are divided into three main categories: biological processes, cellular components and molecular functions. In total, 16,332 unigenes with BLASTX matches were assigned to gene ontology.

Based on NR annotation, 16,332 unigenes were assigned gene ontology (GO) terms. GO-annotated unigenes belonged to the cellular components, molecular function and biological processes clusters and were further divided into 39 subcategories. Cell or cell part, binding and metabolic processes were the vast majority of the categories from each GO cluster (Fig 3). This suggested that the life cycle of *B*. *macrocarpa* is prominently governed by genes related to cellular structure, molecular interaction and metabolism In contrast, among the subcategories with the fewest members were ‘extracellular region part ‘of the cellular components ontology, ‘nutrient reservoir’ of the molecular function ontology and ‘developmental process’ of the biological processes ontology.

**Fig 4:**
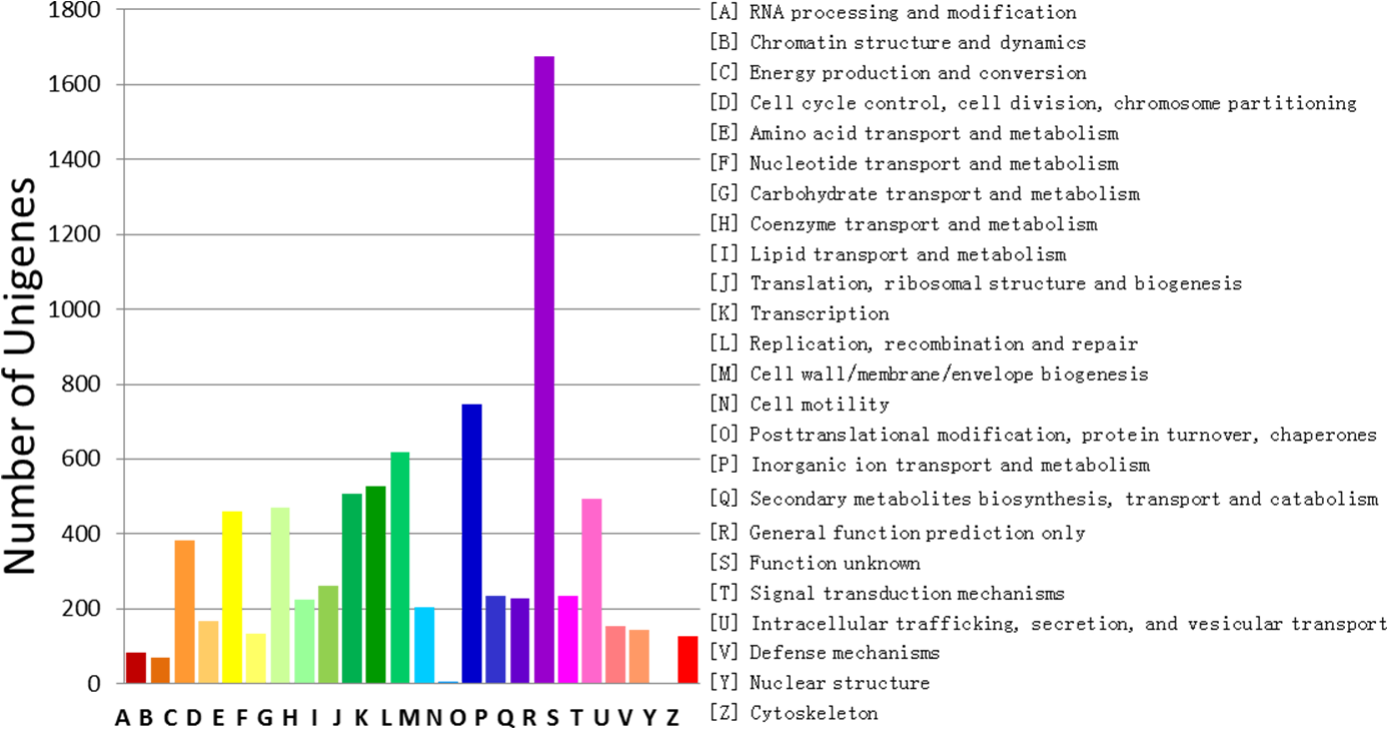
Histogram classifying COG. A total of 14,116 unigenes were grouped into 24 COG categories.

To further evaluate the completeness of our transcriptome library and effectiveness of our annotation process, we also searched the annotated sequences for the genes involved in the clusters of orthologous group (COG) classifications. In total, of 39,179 NR hits, there were 14,116 sequences with a COG classification (Table 3). Among the 24 COG categories, the cluster for ‘General function prediction only’ (20.53%) was the largest group, followed by ‘Posttranslational modification, protein turnover, chaperones’ (9.13%) and ‘Replication, recombination and repair’ (7.57%) clusters, while the category ‘Nuclear structure’ (0.04%) was the smallest group (Fig 4).

Pathway-based analysis helps in further understanding the biological functions of genes. To identify the biological pathways active in *B*. *macrocarpa*, we mapped all the unigenes to the referential canonical pathways in the Kyoto Encyclopedia of Genes and Genomes (KEGG) database. Of the 75,917 unigenes, 7,209 were matched in the databases and assigned to 261 KEGG pathways (Table 3 and S2 Table). Among them, spliceosome pathway (492 members) was the largest group, followed by plant–pathogen interaction pathway (271) and plant hormone signal transduction pathway (246).

### Putative molecular markers

Molecular markers have proven to be valuable tools for various applications in genetics and breeding [17]. Therefore, to develop a novel set of functional simple sequence repeats (SSRs), the *B*. *macrocarpa de novo* assembly transcriptome sequences were mined for the presence of microsatellite motifs using the microsatellite identification tool (MISA). In total, 3,564 sequences containing 4,834 SSRs were identified from 75,917 consensus sequences, with 761 sequences containing more than one SSR (S3 File). The most abundant type of repeat motif were tri-nucleotides (80.39%), followed by di- (14.07%), tetra- (3.23%), hexa- (1.47%) and penta-nucleotide repeats (0.85%)(Table 4). The SSR frequency is dependent on several factors such as genome structure or composition, and arithmetical method for SSR detection and parameters for exploration of microsatellites [18]. In general, it is expected that the frequency of di-, tri-, tetra-, penta- and hexa-nucleotide repeats should simultaneously decrease [19]. However, in *B*. *macrocarpa*, tri-nucleotides were the most abundant SSR type, consistent with reports for lentil, radish, purple sweet potato and other plants [20–22]. Moreover, in mining of SSRs in sugar beet, tri-nucleotides were the most frequently occurring motif type, whereas di-nucleotide repeats were the most abundant SSRs in genomic DNA libraries [23]. Overall, among all types of SSRs, the dominant repeat motif was TGG/CAA (8.45%), followed by GA/TC (6.83%), TGA/TCA (6.77%), GAT/ATC (5.60%) and ATG/CAT (5.33%). The frequencies of SSRs based on number of motifs revealed that SSRs with five tandem repeat motifs (48.45%) were the most common, followed by six tandem repeat motifs (20.96%), and > 10 tandem repeat motifs was least (1.65%; Table 4). Up to now, although some genomic SSRs were available for some wild beets and sugar beet [23–26], there were only a limited number of microsatellite sequences for *B*. *macrocarpa*. The large numbers of potential molecular markers found in our study will be particularly useful for future genetic mapping, parentage analysis, genotyping and resistant breeding of beet species.

**Table 4:** Frequency of occurrence of SSRs in *B*. *macrocarpa* transcriptome.

Motif length	Repeat numbers							Total	%
	5	6	7	8	9	10	>10		
**Di**	-	-	-	353	127	130	70	680	14.07
**Tri**	2160	951	700	64	1	3	7	3886	80.39
**Tetra**	117	39	0	0	0	0	0	156	3.23
**Penta**	34	6	1	0	0	0	0	41	0.85
**Hexa**	33	18	9	1	4	3	3	71	1.47
**Total**	2344	1014	710	418	132	136	80		
**%**	48.45	20.96	14.68	8.64	2.73	2.81	1.65		

### Genes responding to BNYVV infection in *B*. *macrocarpa*

**Fig 5:**
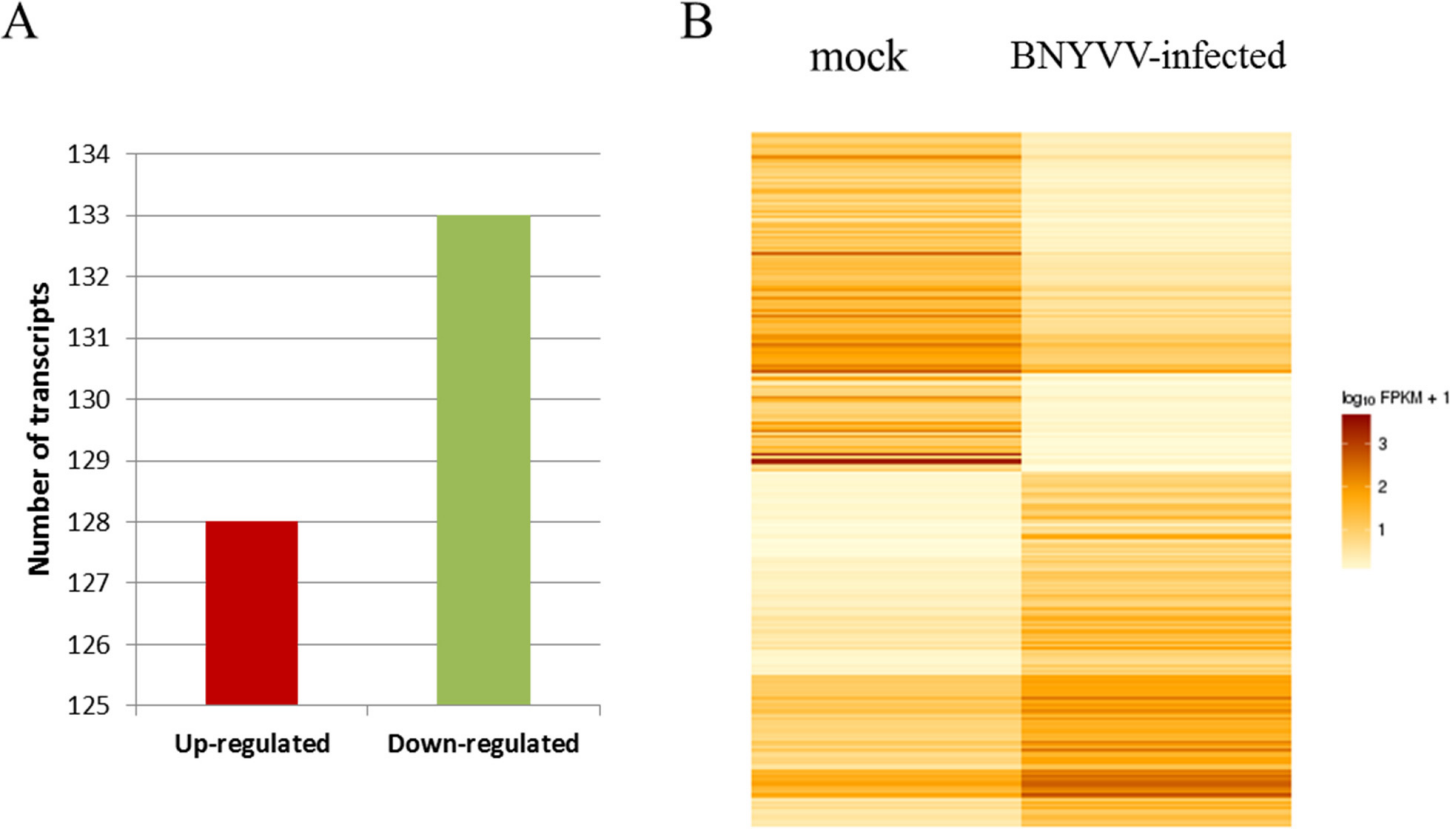
Analysis of differentially expressed unigenes in BNYVV- and mock-infected *B*. *macrocarpa*. (A) Numbers of differentially expressed unigenes between the mock and BNYVV-infected plant libraries. (B) Hierarchical clustering of differential expression profiles for 261 genes between the mock- and BNYVV-infected plant libraries (FDR ≤ 0.05 and absolute value of the log_2_ ratio ≥ 2).

To identify genes showing a significant expression change in *B*. *macrocarpa* upon virus infection, the differentially expressed genes between healthy and BNYVV-inoculated leaves were identified by an algorithm [27] based on the criteria of significance [False Discovery Rate (FDR) ≤ 0.05]. Comparison of gene expression revealed 261 genes differentially expressed between the two samples, including 128 up-regulated and 133 down-regulated genes (Fig 5A). Among the 261 differentially expressed genes, 208 (79.69%) genes were well annotated, whereas the remaining 53 (20.31%) genes had low sequence homology to known sequences in public databases (S2 File), suggesting that they might be putative novel genes in *B*. *macrocarpa* involved in response to BNYVV infection.

RNA-Seq provides a platform for a more sensitive measuring of differences in gene expression than traditional microarray hybridization experiments [28]. We used our RNA-Seq data to analyze the expression of all previously annotated genes, as well as a set of novel transcripts that were uncovered in this study. Fragments per kilobase per million reads (FPKM) values were determined for all genes in each sample, and the resulting data were transformed by first dividing each value for a gene at a particular condition by that gene’s mean FPKM value across two samples and then taking log_10_ of the resulting values. These data were then subjected to hierarchical clustering using Pearson’s correlation coefficient as the distance metric (Fig 5B). This enabled us to determine the similarity in relative change for each transcript in response to BNYVV infection and how these changes were similar or different between transcripts.

### Functional categorization of the identified genes regulated by BNYVV

**Fig 6:**
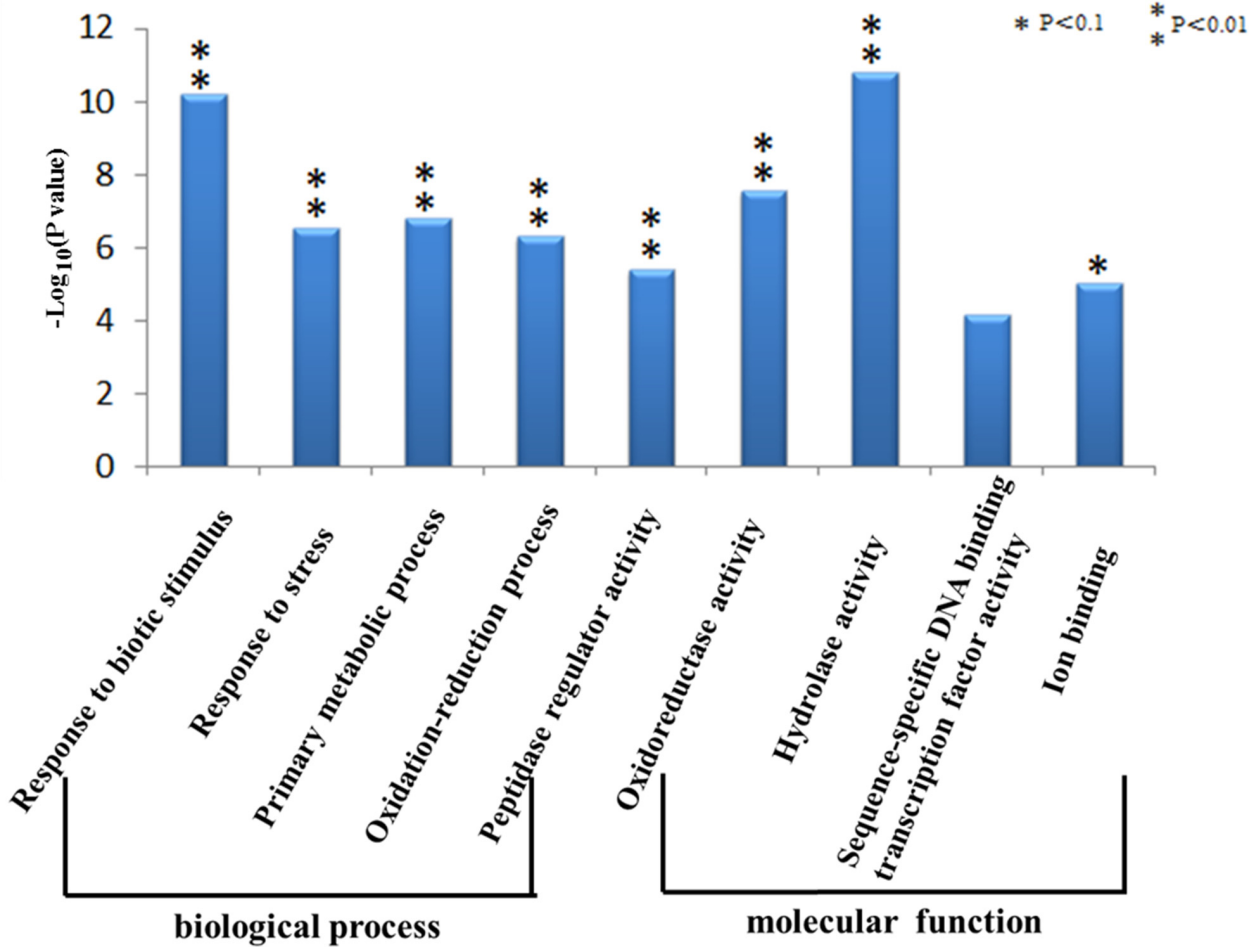
GO enrichment differentially expressed genes between BNYVV-infected and non-infected *B*. *macrocarpa* leaves. The Y-axis is –log_10_ transformation of the P-value calculated in enrichment test.

GO analysis showed that differentially expressed genes in BNYVV-infected *B*. *macrocarpa* were significantly (FDR ≤ 0.01) enriched in two main categories—molecular function and biological process—with four and five functional groups, respectively (Fig 6). The most enriched functional groups of the BNYVV-infected population had altered transcripts in response to biotic stimulus, primary metabolic process, oxidoreductase activity and hydrolase activity.

A common aspect of plant virus or viroid infection shown by expression profiling experiments is the induction of biotic and abiotic stress response genes [29–34]. Consistent with these findings, > 50% of the differentially expressed genes were involved in biotic stress and wound responses, reactive oxygen species (ROS) metabolism, pathogenesis-related (PR) proteins, transcription factors and putative disease resistance genes (S2 File). Plant transcription factors are often involved in response to biotic stimulus [35–39]. During BNYVV infection, regulation of transcription factors and DNA- or RNA-related genes was observed; among them, six were up-regulated and 10 down-regulated (S2 File). The transcription factors, e.g. WRKY and AP2, are regulated by plant hormones such as jasmonic acid, salicylic acid and ethylene as well as by pathogen challenge, and are consistent with the existence of a highly complex regulatory network underlying the physiological response to viral infection [36,39]. In particular, WRKY6 is important for its ability to regulate the expression of defense gene PR-1 in *Arabidopsis* and *N*. *benthamiana* [37]. In addition, the changes of transcription factor genes could be related to expression of BNYVV protein P14 and P31, which are silencing suppressors [6,40] and are both located in nuclei [41].

PR proteins are often triggered during the early response to pathogen attack. In our study, several classes of PR proteins—including PR-1a, PR-2 (beta-1,3-glucanase), PR-3 (chitinase) and PR-5 (thaumatin family proteins)—accounted for nearly 10% of up-regulated defense transcripts in response to BNYVV infection. The expressions of both PR-2 and PR-3 were both induced at a high level by BNYVV in *B*. *vulgaris* and *N*. *benthamiana* [42,43]. This suggests that these genes might have similar expression patterns in response to BNYVV infection, regardless of plant species.

For the primary metabolism, nine genes were up-regulated and 14 down-regulated in BNYVV-infected plants. Most genes involved in ATP-dependent proteolysis, amino acid metabolism and carbohydrate metabolism were down-regulated in BNYVV-infected plants, indicating that protein synthesis and amino acid and carbohydrate metabolism were inhibited by BNYVV infection. A similar effect has been reported for other plants infected by this virus [29,31,32]. The ability of viruses to interfere with these basic host functions offers opportunities not only to probe basic plant functions but also to decipher the molecular events associated with symptom development [44]. Thus, primary metabolism of *B*. *macrocarpa* can be disturbed by BNYVV, which might be correlated with development of systemic symptoms (arrest of growth and yellow mosaic). A previous study in *N*. *benthamiana* showing that suppression of CESA genes and decreases in GA accumulation act in concert to contribute to the stunted growth occuring during BNYVV (RNA_1+2+3+4_) infectious. In addition, altered expressions of genes involved in the RNA silencing pathway (AGO 4, AGO 5,AGO 10, Rnase Ⅲ-like protein) were observed in BNYVV-infected *N*. *bethamiana*, but no substantial difference in the expression levels of putative RNA silencing related genes was found in *B*. *macrocarpa* after infection with BNYVV.These differents may be caused by the diveristy among the host defense systems.

The expansin gene was up-regulated in BNYVV-infected tissues and is an interesting case—it is involved in cellular expansion of cell walls and was found important in determining root phenotype, with a link described between its expression and root hair initiation [45]. Moreover, expansin genes were previously reported as typically up-regulated in infected sugar beet roots and p25 protein-expressing transgenic *Arabidopsis* plants [42,46]. We postulated that the root proliferation observed on rhizomania-infected sugar beet was possibly due to over-expression of the expansin gene.

### Verification of differential gene expression by quantitative real-time PCR (qRT-PCR)

**Fig 7:**
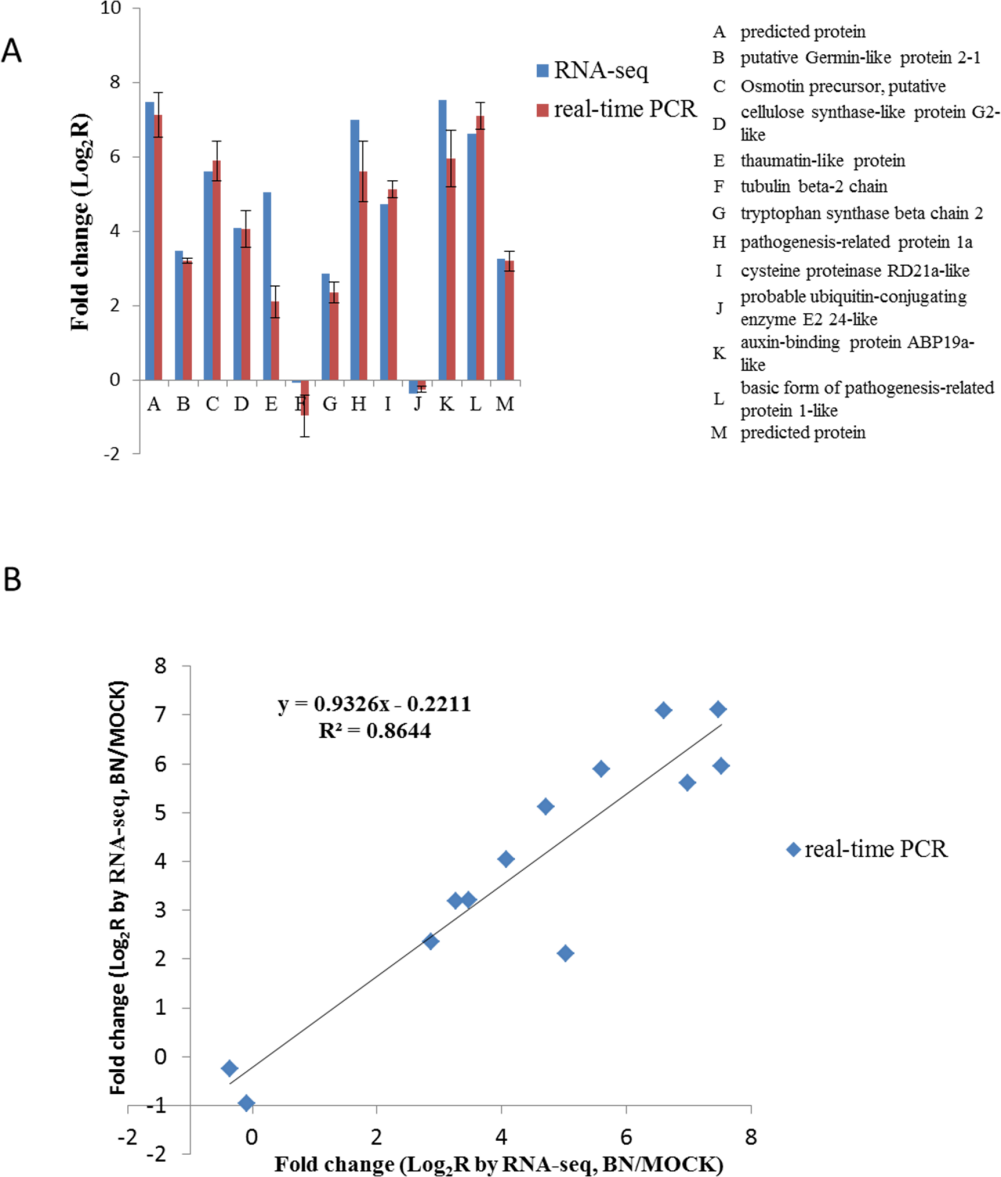
Verification of the relative expression levels of genes by qRT-PCR. (A) Expression patterns of selected *B*. *macrocarpa* genes in response to BNYVV, as determined by qRT-PCR (Red) and RNA-Seq (Blue). The X-axis shows the annotations of selected genes. The Y-axis shows the normalized expression levels of the transcripts. (B) Correlation of the expression ratio of selected genes measured by qRT-PCR and RNA-Seq.

In order to validate the reliability of Solexa sequencing, we compared the expression profiles of BNYVV-infected and non-infected *B*. *macrocarpa* using qRT-PCR. Twenty unigenes with annotations were selected randomly for testing using specific primers (S3 Table, see supporting information). There were 13 of 17 unigenes (76.47%) examined with the same expression profiles as deduced from the original Solexa sequencing (Fig 7A). Inconsistencies among the remaining four genes could be artificially caused by mutations with primer sites or possibly a lower sensitivity of qRT-PCR than RNA-Seq [47,48]. Nevertheless, a high correlation (R^2^ = 0.8644) between RNA-Seq and qRT-PCR (Fig 7B) suggests that the transcriptome data is reliable and reflects the actual direction of gene expression in response to BNYVV infection.

## Conclusion

This study represents the first application of Illumina sequencing technology to obtain the transcriptome of *B*. *macrocarpa* challenged with BNYVV at an unprecedented depth (10.52 Gb) and produced 75,917 assembled unigenes with 39,372 unigenes obtaining annotation. These findings provide a substantial contribution to existing sequence resources for wild beet, and will likely accelerate research on the sugar beet resistance mechanism to BNYVV. Comparative transcriptome analysis between BNYVV-infected and control plants revealed significant differences in gene expression. Although the molecular functions of some genes and their associated pathways remain largely unknown, this study provides valuable information on the role of the differentially expressed genes in response to BNYVV infection. Future functional analysis of these potential virus defense genes is expected to aid a better understanding of the molecular mechanisms of pathogen-defense in beet. Furthermore, the large number of transcripts and molecular markers obtained in this study offers a strong basis for future genomic research on beets.

## Materials and Methods

### Plant, virus inoculation and detection

*Beta macrocarpa* plants were grown in a controlled-environment chamber at 24 ± 1°C under a 16/8 h light/dark regimen. BN (RNAs 1+2+3+4+5) was a mixture of total RNAs from the BN3 (RNAs 1+2+3) inoculated leaves of *Tetragonia expansa* and *in vitro* transcripts of RNA4 and RNA5. Virus inoculum supplemented with an equal volume of inoculation buffer (50 mM glycine, 30 mM K_2_HPO_4_, 1% bentonite and 1% celite at pH 9.2) was rubbed onto *B*. *macrocarpa* leaves as described previously [49].

Inoculated or uninoculated upper leaves from BNYVV-infected and mock-infected *B*. *macrocarpa* at 15 day post inoculation (dpi) were collected and homogenized in liquid nitrogen for protein extractions with equal volumes of gel loading buffer (100 mM Tris base, pH 6.8; 20% glycerol; 4% SDS; 200 mM β-mercaptoethanol; 0.2% bromophenol blue). For western blot, BNYVV coat protein specific polyclonal antibodies (obtained from rabbit immunized with purified BNYV virions) were applied at a dilution of 1:500. Alkaline phosphatase conjugated to goat anti-rabbit antiserum (Sigma, USA) was used as secondary antibody at a dilution of 1:5000.

### cDNA library preparation and deep sequencing

Total RNAs were extracted from BNYVV-infected and non-infected *B*. *macrocarpa* using TRIZOL reagent (Takara, Dalian, China) according to the manufacturer’s instructions. Integrity and size distribution were checked with Bioanalyzer 2100 (Agilent Technologies, USA). The RNA pool was prepared by mixing together equal quantities of five RNA samples per group. cDNA library preparation and Illumina-Solexa sequencing were performed as previously described. The normalized cDNA libraries were sequenced using an Illumina HiSeq 2000, and all obtained data were submitted to the NCBI database Short Read Archive.

### Analyses of Illumina sequencing results

Raw sequencing reads were quality trimmed, and dirty raw reads (i.e. reads with adaptors or reads with unknown nucleotides > 5% low quality reads) were discarded. Processed reads were assembled using CAP3 software with default parameters [16]. The overall assembly was performed using the combined sequence data for both the BNYVV-infected and non-infected samples. The contigs and singletons were generally referred to as unigenes. Subsequently, unigenes were subjected to BLASTX similarity search against the NCBI NR protein and Swiss-Prot databases with a significant threshold of E-value ≤ 10^−5^. Functional categorization by GO terms was performed using Blast2GO software [50]. COG and KEGG pathway annotations were performed using Blastall software against the COG [51] and KEGG databases [52], respectively.

### SSR analysis

All assembled cDNA contigs from both the infected and control libraries were used for identification of SSRs. All types of microsatellites from di- to hexa-nucleotides were detected using MISA software [53] (http://pgrc.ipk-gatersleben.de/misa/) with a minimum repeat number of eight, five, five, five and five for di-, tri-, tetra-, penta- and hexa-nucleotide microsatellites, respectively. A Perl script was designed to allow the identification and characterization of microsatellites in a comparative genomic context.

### Evaluation of different expressed genes

FPKM was used to evaluate expressed value and quantify transcript levels. We used FDR ≤ 10^−5^ as the threshold and log_2_ ratio (BNYVV-infected/ non-infected) ≥ 1 to judge the significance of gene expression difference. For enrichment analysis, we mapped all differentially expressed genes to terms in GO and compared this with the genome background (the whole *B*. *macrocarpa* transcriptome in this study).

### qRT-PCR analysis

To confirm the results of transcriptome sequencing analysis, the relative mRNA expression levels of several randomly selected genes in RNA from mock- and BN-infected leaves were evaluated. Three RNA samples were detected for each group; whereas one was the same as that used for RNA-Seq analysis, the other two replicates were derived from different plant samples. qRT-PCR was performed in 96-well plates using the CFX96 real-time PCR detection system (Bio-Rad, Hercules, CA, USA) with the following temperature program: 95°C for 15 s, followed by 40 cycles of 95°C for 15 s, and then annealing at 60°C for 30 s. For relative quantification of mRNA, 1 μg of total RNA was extracted from leaves, treated with DNaseI (Takara) and reverse transcribed following the manufacturer’s instructions. Each reaction mixture consisted of 1 μl of cDNA, 7 μl of SsoFast EyaGreen Supermix (Bio-Rad), 1.5 μl (3 pmol/μl) of both forward and reverse primers, and 3 μl of PCR-grade water, as recommended by the manufacturer (Takara). Each reaction included amplification of PP2A transcripts, which provided an internal reference. All primers used in this study are listed in S3 Table. All PCR experiments were performed in triplicate and the results were calculated using the CFX Manage Version 1.6 software (Bio-Rad, Hercules, CA, USA) with the default parameters.

## Supporting Information

S1 File Summary of functional annotations of non-redundant unigenes, showing the results of searches of reference sequences using BLASTX against the NCBI NR, GO and KEGG databases with a cut-off E-value of 10^−5^. (XLSX)

S2 File Differentially expressed transcripts between healthy and BNYVV-infected samples. (XLSX)

S3 File Frequency of SSRs occurred in *B*.*macrocarpa*. (XLSX)

S1 Table Summary of data production. (DOCX)

S2 Table Unigenes KEGG pathway analysis. (XLSX)

S3 Table Primers used in qRT-PCR. (XLSX)
