# Supplementary material for: Transcriptome Analysis of Beta macrocarpa and Identification of Differentially Expressed Transcripts in Response to Beet Necrotic Yellow Vein Virus Infection
Source: PLoS One. 2015 Jul 21;10(7):e0132277. doi: 10.1371/journal.pone.0132277 (PMC4719419; doi:10.1371/journal.pone.0132277)
Supplement: S1 Table — (DOCX) [file pone.0132277.s004.docx]

**Table S1** Summary of data production

| Sample | Raw reads | Raw bases | Clean reads | Clean bases | Clean data rate |
| --- | --- | --- | --- | --- | --- |
| BN | 66,927,046 | 6,692,704,600 | 59,903,306 | 583,0613,062 | 87.12% |
| Mock | 54,342,684 | 5,434,268,400 | 48,279,346 | 469,3645,595 | 86.37% |
